# Sparse Convolutional Neural Networks for Genome-Wide Prediction

**DOI:** 10.3389/fgene.2020.00025

**Published:** 2020-02-06

**Authors:** Patrik Waldmann, Christina Pfeiffer, Gábor Mészáros

**Affiliations:** ^1^ Department of Animal Breeding and Genetics, The Swedish University of Agriculutural Sciences, Uppsala, Sweden; ^2^ Division of Livestock Science, University of Natural Resources and Life Sciences Vienna (BOKU), Vienna, Austria

**Keywords:** genomic selection, machine learning, deep learning, dominance, QTL, livestock breeding

## Abstract

Genome-wide prediction (GWP) has become the state-of-the art method in artificial selection. Data sets often comprise number of genomic markers and individuals in ranges from a few thousands to millions. Hence, computational efficiency is important and various machine learning methods have successfully been used in GWP. Neural networks (NN) and deep learning (DL) are very flexible methods that usually show outstanding prediction properties on complex structured data, but their use in GWP is nevertheless rare and debated. This study describes a powerful NN method for genomic marker data that can easily be extended. It is shown that a one-dimensional convolutional neural network (CNN) can be used to incorporate the ordinal information between markers and, together with pooling and *ℓ*_1_-norm regularization, provides a sparse and computationally efficient approach for GWP. The method, denoted CNNGWP, is implemented in the deep learning software Keras, and hyper-parameters of the NN are tuned with Bayesian optimization. Model averaged ensemble predictions further reduce prediction error. Evaluations show that CNNGWP improves prediction error by more than 25% on simulated data and around 3% on real pig data compared with results obtained with GBLUP and the LASSO. In conclusion, the CNNGWP provides a promising approach for GWP, but the magnitude of improvement depends on the genetic architecture and the heritability.

## Introduction

Extraction of valuable information from big data is a critical endeavour in different disciplines of genomics and bioinformatics (Fan et al., 2014). A major direction of machine learning is to develop efficient algorithms for analysis and model prediction of large scale data sets (Ghahramani, 2015; Jordan and Mitchell, 2015). There are many instances of the effective use of machine learning in genomic prediction, genome-wide association studies and different types of DNA-sequence analysis (Okser et al., 2014; Libbrecht and Noble, 2015).

Artificial neural networks (NN) are multifaceted methods that recently have attracted much attention because of their superior prediction characteristics (LeCun et al., 2015). The input layer of a NN is connected to an output layer, either directly or through one or several hidden layers of interconnected neurons. The number of hidden layers determines the depth of a NN, and the width depends on the number of neurons of each layer. Deep neural networks (DNNs) are NNs with several hidden layers. Fast optimization algorithms are used to iteratively perform forward and backward passes for minimization of a loss function and to learn the weights and biases of the layer. The activation functions are applied to the current values of the weights at each layer in the forward pass. The final result of a forward pass is new predicted outputs. The backward pass computes the error derivatives between the predicted outputs and the real outputs. These errors are then propagated backwards updating the weights and calculating new error terms for each layer. Iterative repetitions of this process is denoted back-propagation (Rumelhart et al., 1986). Stochastic gradient descent (SGD) is the most common technique to train parameters in NNs. Several variants of SGD have been developed and some of them adapts the learning rate automatically throughout the course of learning (Goodfellow et al., 2016).

The multilayer perceptron (MLP) is the most basic NN and its minimum configuration consists of three layers of nodes: an input layer, a hidden layer, and an output layer (Rosenblatt, 1962). Except for the input nodes, each node is a neuron that uses a nonlinear activation function. The MLP can be seen as a hierarchical mathematical function mapping some set of input values to output values *via* many simpler functions. Normally, the nodes are fully connected between layers and therefore the number of parameters quickly increases to huge numbers with a considerable risk of overfitting. There are several techniques to avoid overfitting in NN (Theodoridis, 2015).

The convolutional neural network (CNN) technique is built around sharing of weights and is influenced by the structural architecture of the human visual system. CNNs are based on ideas that utilize local connectivity between neurons and hierarchically organized transformation of the input (Min et al., 2017). Nodes form groups of *d*-dimensional arrays known as feature maps. Each node in a given map receives inputs from a certain window area of the previous layer, which is referred to as its receptive field. The convolution operation results in a much sparser NN than the MLP. The first CNN was proposed by LeCun et al. (1989) and the technique has become popular in the analysis of structured data, for example in speech recognition and image analysis.

Gianola et al. (2011) suggested Bayesian ridge regression based regularization to prevent over-fitting in feed-forward NNs and showed that this improved prediction accuracy of traits in Jersey cows and wheat compared to standard linear models. The approach was further improved by replacing the computationally demanding Levenberg-Marquardt training algorithm with back-propagation (Ehret et al., 2015). Bellot et al. (2018) compared a few MLPs and CNNs with each other on large human SNP and phenotype data. They found that, in general, the performance of CNN was comparable to that of regularized linear models, but suggested that more research was needed to adapt the CNN methodology to genetics-based studies. This conclusion is further supported by conflicting results between different methods in a recent review (Pérez-Enciso and Zingaretti, 2019).

The purpose of this study was to further evaluate the CNN methodology within the GWP framework. Tuning of the structure and hyper-parameters of the CNN was performed with Bayesian optimization (Mockus, 1975). Moreover, the importance of sparsity inducing regularization was evaluated. The predictive properties of the CNN models were evaluated on both simulated data with additive and dominance genetic effects and real pig data. To our knowledge, this is the first time a 1d CNN is combined with *ℓ*_1_-norm regularization, Bayesian optimization and ensemble prediction in a GWP setting.

## Methods

### Neural Networks

The multilayer perceptron (MLP) is defined as

(1) y=f(X;θ)

where function *f*(·) maps an input *X* (genomic markers) of dimension *n*×*p* to an output *y* (phenotype) of dimension *n*×1, and learns the parameters θ that result in the best function approximation. This model is also known as a feedforward network because information flows from *X* through *f*(·) to *y* without any feedback connections (LeCun et al., 2015). The MLP usually consists of several functions expanded over each other, for example three functions *f* ^(1)^(·), *f* ^(2)^(·), and *f* ^(3)^(·) which results in the connected chain *f*(X) = *f* ^(3)^(*f* ^(2)^ *f* ^(1)^(*x*))) where *f* ^(1)^(·) is referred to as the first layer, *f* ^(2)^(·) the second (hidden) layer and the final layer is called the output layer. The length of the chain defines the depth of the NN. Each layer consists of one, or usually more, units called nodes that act in parallel. The number of nodes per layer determines the width of the model.

With only input and output layers, model (1) is equivalent to a linear model. However, it is straightforward to apply a nonlinear transformation *ϕ*(·) to the input and thereby obtain a more flexible model

(2) $$y=f(X;\theta ,W,b)=\phi {\left(X;\theta \right)}^{T}W+b$$

where *θ* are the parameters of the (non)-linear activation functions, *W* are the weight parameters that map from *ϕ*(*X*) to the output, and *b* is the bias parameter that captures the overall deviation from zero. The activation functions are usually applied to the affine transformation *z *= *W* ^*T*^ *X*+*b* of the hidden layers. There is a large number of activation functions to choose from. Unfortunately, there is no activation function that works best in all situations. Hence, a trial-and-error approach seems to be the most viable solution.

The rectified linear unit (ReLU) has become a popular default activation function and is defined as *ϕ*(*z*) = max{0,*z*}.The only difference between a linear function and ReLU is that the latter outputs zero across half of its domain. This makes the derivatives through a ReLU remain large and consistent when the function is active. However, it should be noticed that the ReLU is not differentiable at *z =* 0, but this seems to have relatively small effects on gradient-based algorithms in practice (Goodfellow et al., 2016). There are several variants of the ReLU function, for example the leaky ReLU, parametric ReLU and maxout.

Training of the MLP parameters is usually performed with a gradient-based technique in order to minimize a loss (i.e., cost) function

(3) $$\underset{\theta ,W,b}{\arg\min}\;J(\theta ,W,b)$$

which is equivalent to minimization of the negative log-likelihood -log *p*(*y*|*X*). The characteristics of the output variable determine which cost function and output activation function to use. For continuous output variables the modeling approach is regression where it is most common to use mean squared error (MSE) cost following

(4) $$\underset{f}{\arg\min}||y-\hat{f}(X;\theta ,W,b)|{|}_{2}^{2},$$

where $||\cdot |{|}_{2}^{2}$ denotes the squared Euclidean loss and $\hat{f}(X;\theta ,W,b)$ is the predicted output. Another popular cost function for continuous output is the mean absolute error (MAE) which is based on the *ℓ*_1_-norm, ||⋅||_1_, and therefore considered to be less sensitive to outliers. For categorical outputs the problem becomes classification. Loss functions for categorical data include the logistic, the cross entropy, and the hinge loss. The two former loss functions are continuous and convex, and therefore can be optimized with gradient descent methods. The hinge loss is used in Support Vector Machines, but is not convex and needs to be minimized with subgradient descent methods and was not further investigated in this study. The choice of loss function is closely connected with the choice of output activation function. In regression, it is common to use the linear output activation, whereas binary classification often use the sigmoid function and multinomial classification uses the softmax function.

### Convolutional Neural Networks

Convolutional neural networks are a specialized form of NN for analysis of input data that contains some form of spatial structure (Goodfellow et al., 2016). Examples include time-series data which is one-dimensional, image data which is two-dimensional and video data which is three-dimensional. A CNN consists of one or several convolutional layers of the form *g* = *C_K_*(*f*) which acts on a *p*-dimensional input *f*(*x*) = (*f*_1_(*x*), …, *f_p_*(*x*)) by applying a set of filters (also referred to as kernels) *K*=(*ĸ_l',l_*) for *l* = 1,…, *q* and *l'* = 1,…, *p* together with a possible non-linear activation *ϕ*

(5) $$g_{l}(x)=\phi \left(\sum_{l^{\prime }=1}^{p}\left(f_{l^{\prime }}*\kappa_{l^{\prime },l})(x\right)\right)$$

producing a *q*-dimensional output *g*(*x*) = (*g*_1_(*x*),…,*g_q_*(*x*)), often referred to as a feature map. Here, each convolution operation is over discrete univariate index variables and therefore becomes $(f*\kappa )(x)=f_{l^{\prime }}\left(x\right)\kappa_{l^{\prime },l}$. A discrete convolution can be interpreted as a multiplication of the input vector with a Toeplitz matrix with the kernel repeated on the diagonal. Hence, for a univariate discrete convolution, each row of the matrix is constrained to be equal to the row above, but sequentially shifted by one element (Goodfellow et al., 2016). In the first layer of a genetic application, *l*' will refer to SNP index or chromosome position, *q* to the size of the window to slide over the chromosomes, and *x* the genotype value. In a fully connected MLP, every output unit interacts with every input unit. However, in a CNN, the interactions are sparse because the filter is normally set to be of smaller size than the input. This means that fewer parameters need to be calculated and stored which improves computational efficiency.

The convolution layer expands the parameter space considerably and therefore is often followed by a pooling layer *g = P*(*f*) that replaces the full output with a summary statistic of the neighborhood *V*(*x*) of the convolution

(6) $$g_{l}(x)=P(f_{l}(x^{\prime }):x^{\prime }\in V(x))$$

where *x* ^′^ refers to the set of values from the neighborhood to consider for the pooling function. There are several pooling functions *P* that can be used. The max pooling function provides the maximum output of the convolution neighborhood. Other pooling functions reports the weighted average or some norm regularized measure. The size of the pooling function is typically smaller than the size of the convolution which reduces the parameter space even further. Sometimes it is also beneficial to systematically skip some positions of the filter kernel in order to reduce the precision. A powerful approach for this is known as strided convolution. A stride length of two means that only every second input is sampled, which also leads to a halved number of parameters. Another important feature of any CNN is the ability to zero pad the input to make it wider. Without this feature the width of the input shrinks by one unit for each layer and allows for independent control over the kernel width and the output size. Equal padding means that the number of zeros is the same on both sides of the input data. Before the output layer is reached, it is necessary to flatten the convolution and it is also useful to regularize these weights with the *ℓ*_1_-norm to prevent over-fitting. Regularization can also be obtained using dropout, where a random fraction of the nodes (i.e., weights) in a layer are set to zero. The surviving nodes have to stand in for those that are omitted, which produces a form of regularization that has been shown to be effective in preventing over-fitting. In the CNN layer dropout is usually performed by treating filters as units and all weights belonging to the same filter are set to zero. Pérez-Enciso and Zingaretti (2019) provides further explanation and illustration of CNNs.

### Bayesian Optimization of Hyperparameters

Bayesian optimization (BO) forms a set of powerful tools that allows efficient black-box optimization and has general applications in a broad spectrum of fields (Shahriari et al., 2016). It is applicable in situations where one does not have a closed-form expression for the objective function and its hyperparameters *f*(*X;γ*), but where it is possible to perform noisy evaluations of this function at sampled values of *γ*. BO is particularly useful when the evaluations of *f*(*X*;*γ*) are demanding, when derivatives are difficult to obtain, or when the optimization problem is non-convex. BO is a probabilistic sequential approach with two key steps. First, it uses the entire sample history to update a posterior distribution over the unknown *f*(*X*;*γ*). Second, it uses an acquisition function α to trade off between exploration and exploitation when selecting the points of *γ* at which to evaluate next. The optima are located where the uncertainty (the variance) in the objective function is large (exploration) and/or where the function value of the model (the mean) is high (exploitation).

One common BO approach is to assume that *f*(*X*;*γ*) is distributed according to a Gaussian process prior:

(7) $$f(X;\gamma )\sim \text{GP}(m(X;\gamma ),C((X;\gamma_{i}),(X;\gamma_{j}))$$

where the properties of the resulting distribution are completely detemined by the mean function *m*(*X*;γ) and the covariance function *C*((*X*;*γ*_*i*_),*X*;*γ*_*j*_), and it is assumed that the negative of the test MSE observations are normally distributed:

(8) $$-\text{MSE}\sim N(f(X;\gamma ),\sigma^{2}I).$$

Given this generative model, the posterior over functions will be the acquisition function *α* which determines the next point to evaluate *via* a proxy optimization:

(9) $$\gamma_{t+1}=\arg\max\alpha (\gamma_{1:t}).$$

One useful acquisition function that fits well with the GP approach is the upper confidence bound (UCB) (Srinivas et al., 2010)

(10) $$\alpha_{UCB}=\mu (\gamma_{1:t})+\beta \sigma (\gamma_{1:t}),$$

where *μ* is the mean and σ the standard deviation of *f*(*X*;*γ*_1:*t*_), respectively. ***β*** is a tunable hyperparameter that determines the trade-off between exploration and exploitation. The BO is run for *T* number of iterations.

### Model Averaged Ensemble Predictions

The stochastic sampling of mini-batches in SGD based algorithms introduces uncertainty that leads to parameter fluctuations between iterations (Waldmann, 2018). In NN, poor generalization behavior of the SGD has been observed in practice (Hardt et al., 2015). Ensemble methods use multiple learning algorithms, or replicates of the same algorithm on stochastic manipulations of the data, to obtain better predictive performance than could be obtained from any of the algorithms alone. It is possible to obtain model averaged (MA) estimates by averaging over ensemble predictions. In this study, *S* replicates of the BO were run and the hyperparameters *γ* were extracted from the model with lowest test MSE, which were then averaged to obtain *γ*_MA_. After that, the CNN was re-run *S* times with fixed *γ*_MA_ (without BO), and test predictions were calculated for each of these runs, and finally averaged to obtain MA predictions

(11) $${\hat{y}}_{\mathrm{MA}}=\frac{1}{S}\sum_{s=1}^{S}\hat{f}{\left(X;\theta ,W,b,\gamma_{\mathrm{MA}}\right)}_{s}$$

which was used to calculate minimum test MSE_MA_.

## Data

### Simulated Data

The primary data was created for the QTLMAS2010 work-shop and consists of 3,226 individuals structured in a pedigree with 5 generations (Szydłowski and Paczyńska, 2011). The pedigree is founded on 20 individuals (5 males and 15 females). Each female is mated once giving birth to approximately 30 progeny. A neutral coalescent model was used to simulate the SNP data where the genome is made up of five autosomal chromosomes each with a length of 100 Mbp. This procedure resulted in 10,031 markers, including 263 monomorphic and 9,768 biallelic SNPs.

The continuous quantitative trait was created from 37 QTLs, including 9 controlled genes, and 28 random genes. The controlled QTLs included two pairs of epistatic genes with no individual effects, three maternally imprinted genes and two additive major genes with effects of -3 and 3. The additive genes are positioned at SNP indices 4,354 and 5,327, whereas the major epistatic locus is at SNP 931. The random genes were selected among the SNPs. Their effects were sampled from a truncated normal distribution and designated to be a QTL if the absolute value of the additive effect was smaller than 2. The QTLs were surrounded by 19–47 polymorphic SNPs (MAF > 0.05) located within 1 Mb distance from the QTL. 364 SNPs display moderate to high linkage disequilibrium (LD) with the QTLs.

In addition to the original data simulated by Szydłowski and Paczyńska (2011), one dominance locus was placed at SNP number 9212 by assigning an effect of 5.00 to the heterozygote and a value of 5.01 to the upper homozygote. One over-dominance locus was created at SNP 9404 by allocating an effect of 5.00 to the heterozygote, and an effect of -0.01 to the lower homozygote and 0.01 to the upper homozygote. Finally, by allocating a value of -5.00 to the heterozygote, an effect of -0.01 to the lower homozygote and 0.01 to the upper homozygote, one under-dominance loci was generated at SNP id 9602. The effects of the genotypes of these new QTLs were added to the original phenotype values. Minor allele frequency (MAF) cleaning was performed at the 0.01 level, so the final sample of SNPs with 0,1,2 coding was 9723. The phenotype values were mean standardized. The SNP data was normalized to mean zero and variance one over each SNP. Data from individual 1 to 2,326 was used as training data and from individual 2,327 to 3,226 (the 5th generation) as validation data.

### Real Data

For evaluation of the CNN methodology within the GWP framework, 808 Austrian Large White and Landrace sows with high-density genotypes were used. Genotyping was carried out using Illumina PorcineSNP60 BeadChip (Illumina, San Diego CA, USA). SNPs were quality controlled using the software package PLINK 1.9 (Purcell et al., 2007), excluding SNPs if MAF was lower than 0.01 and if SNPs deviated strongly from Hardy-Weinberg equilibrium (< 10^-9^). After quality control 50,174 SNPs remained, recoded to additive genotype format (0,1 or 2) with PLINK using −−recode A. A total of 207,488 genotypes were missing and therefore imputed with FImpute using pedigree information (Sargolzaei et al., 2014).

Most of the genotyped sows had repeated phenotypic information for the trait number of live born piglets. For the evaluation based on real data, the phenotypic records were averaged over individuals. The number of measurements ranged from 1 to 9 with a median value of 4. The heritability for number of live born piglets were 0.16 ± 0.04 using pedigree evaluation based on 7,940 pigs. For comparative purposes, we used the breeding values (EBVs) from the pedigree evaluation as one trait (808 records) and the real measurements as another trait (702 records). For cross-validation (CV), data was partitioned into eight folds with each training data of 707 individuals and each test data containing 101 individuals for the EBV trait and into seven folds for the real measurements.

## Implementation

Nowadays, there are several software packages designed for deep learning. In this study, the R interface to Keras (Chollet et al., 2017) with the Tensorflow back-end was used. As a base model, a 1-dimensional CNN with an MSE loss function was implemented. The input (SNP markers) was connected to a 1d CNN layer. This layer was followed by max pooling and flatten layers. At the end, a 1-unit dense output layer with linear activation and *ℓ*_1_-norm regularization of the weights was connected to the output (phenotypic values). The adaptive moment estimation (ADAM) optimizer, which adapts the decay of the learning rate using moving average and bias-correction, was chosen for all analyses (Kingma and Ba, 2014; Waldmann, 2018). The R package rBayesianOptimization (Yan, 2016) was used for UCB based BO using the minus of the validation MSE as score value since this package cannot perform minimization directly. The R-code for this model is denoted CNNGWP and can be found at github (Waldmann, 2019).

For comparison, the simulated and real data were also analyzed with the genomic best linear unbiased prediction (GBLUP) and Bayesian LASSO (BLASSO) implemented in the using default settings in the R-package BGLR Pérez and de los Campos, 2014).

## Results

### Simulated Data

Together, the model and optimizer have at least 10 γ that need to be tuned. BO of all these *γ* proved to be difficult, probably because of structural dependencies in the NN that led to flat ridge-like structures in the MSE surface. Hence, after some initial evaluations against validation data where some of the *γ* were fixed, the following parameters of the 1d CNN layer were fixed: linear activation function, equal zero padding, and strides of two, because they yielded relatively low and overall stable errors. Interestingly, ReLU activation and 1d spatial dropout showed no decrease in MSE when applied to this layer, and were for this reason not used. The pool size of the max pooling layer should be smaller than the kernel size and therefore was set to two. Moreover, the learning rate of the ADAM optimizer was set to 0.00025 after visual inspection of validation MSE against epoch number of several plots. Too high a learning rate can result in a bumpy MSE trajectory that may miss the minimum, whereas too low a learning rate will require a huge number of epochs to reach the minimum. One epoch refers to a learning pass over all data points (i.e., individuals) and therefore the number of iterations within one epoch depends on the batch size. The batch size was fixed at 48, which is the integer part of the square root of the number of observations in the training data. The maximum number of epochs was set to 250.

This set up resulted in three *γ* that needed to be tuned with BO, namely, the number of filters of the CNN layer (filter), the size of the convolution window (kernel), and the regularization parameter *λ* of the *ℓ*_1_-norm in the output layer. The initial bounds of *γ* were set to [20,100], [10,50], and [0.1,1.0] for the filter, kernel, and *λ*, respectively. It is well-known that SGD and related algorithms suffer from numerical instability and statistical inefficiency, and it has been suggested that averaging of parameters induces stability and results in lower generalization error (Toulis et al., 2016). Hence, in order to obtain a reasonable compromise between computing time and statistical accuracy, the number of iterations in the BO *T* and the number of replicates of the BO and MA *S* were set to 40, 25, and 25, respectively. This resulted in the following *γ*_MA_ estimates from the BO: filter = 64, kernel = 27, and *λ* = 0.571. The type of layers and the number of parameters of the optimized model are outlined in **Table 1** based on the summary() function in Keras. The CNN was re-run 25 times with these hyperparameters to obtain ${\hat{y}}_{\mathrm{MA}}$ which resulted in a final test MSE_MA_ of 62.34. Note that it is not possible to do MA over the weights in the CNN layer because the filters may pick up different QTLs at different runs. Hence, each of the 25 runs was treated as different prediction models. The GBLUP and BLASSO produced mean MSE over folds of 88.42 and 89.22, respectively (**Table 2**). This implies that the improvement for the simulated data was 29.5% and 30.1%, respectively.

**Table 1:** Layer name and type, output data shape of the layer, and number of trainable parameters per layer for the model with the Bayesian optimized model averaged hyperparameters using the simulated data.

Layer (type)	Output shape	# Parameters
Conv1d (Conv1D)	(None, 4862, 64)	1792
Maxpooling (MaxPooling1D)	(None, 2431, 64)	0
Flatten (Flatten)	(None, 155584)	0
Out (Dense)	(None, 1)	155585
Total		157377

The model description is based on the output from the summary() function in Keras. None is an internal Keras representation which means that the mini-batch size is a variable to be set in the consequent fit() function.

**Table 2:** Test mean squared error (MSE) for CNNGWP, GBLUP, and BLASSO for each of the three data sets.

Data set	CNNGWP	GBLUP	BLASSO
QTLMAS2010	62.34	88.42	89.22
Pig EBV	0.301	0.305	0.307
Pig phenotype	3.51	3.64	3.61

**Figure 1:**
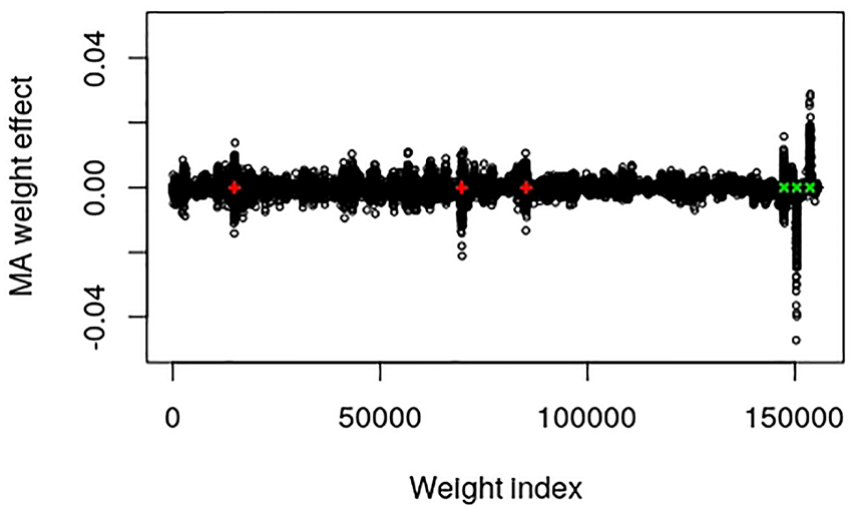
Plot of model averaged weight effects against weight index in the output layer of the simulated QTLMAS2010 data. The true major additive (including the additive part of the epistatic) QTLs are marked in red + and the dominance QTLs are in green ×.

The output layer of this data set contains 155,584 weights and 1 bias parameter. Dividing the number of weights by the number of SNPs in the input layer resulted in a factor that can be used to map the SNP index to approximate positions of the weight index, i.e., 155,584/9723 = 16.002. The epistatic locus mainly contains an additive part that after multiplication with the factor should be found at weight index 14,898. The two major additive SNPs should be positioned around weight indices 69,673 and 85,243, whereas the dominance, over-dominance and under-dominance loci should be at positions 147,410, 150,483 and 153,651, respectively. **Figure 1** provides a plot of the MA weight effects of the output layer against weight index with the five major true QTL positions marked. It is interesting to note that the positions are mapped very well. Moreover, the additive weight effects are centered around zero whereas the dominance effects deviate from zero.

### Real Data

After some initial evaluations against validation data of each of the folds, it was found that the model used on the simulated data could be used also on the real data with the following changes. The learning rate of the ADAM optimizer was changed to 0.00001 for the EBV trait and 0.000025 for the real trait. The batch size was changed to 26 for the EBV trait and to 25 for the real trait, whereas the maximum number of epochs were changed to 150 and 50 for the EBV and real trait, respectively. For BO of the EBV trait, the initial bounds of *γ* were altered to [5,50], [5,50], and [0.002,0.1] for the filter, kernel, and *λ*, respectively. For the real trait, these bounds were set to [5,60], [5,60], and [0.5,5.0] for the filter, kernel, and *λ*, respectively. *T* and *S* were set to 40 and 25 for each of the folds of both real data sets. The BO resulted in *γ*_MA_ estimates: filter = 41, kernel = 40, and *λ* = 0.0071 for the EBV trait. These hyperparameters resulted in a final test MSE_MA_ of 0.301 for the EBV trait. The GBLUP and BLASSO produced mean MSE over folds to be 0.305 and 0.307, respectively. Hence, the improvements of the CNNGWP compared to the GBLUP and BLASSO were 1.3% and 2.0%, respectively. The model characteristics for the EBV trait is summarized in **Table 3**. For the real trait, the BO resulted in *γ*_MA_ estimates: filter = 25, kernel = 34, and *λ* = 2.54 which produced a MSE_MA_ of 3.51. The GBLUP and BLASSO produced mean MSE over folds of the real trait of 3.64 and 3.61, respectively (**Table 2**). This implies that the improvement for the real phenotype was 3.6% and 2.8%, respectively. The model characteristics for the real trait is summarized in **Table 4**.

**Table 3:** Layer name and type, output data shape of the layer, and number of trainable parameters per layer for the model with the Bayesian optimized model averaged hyperparameters using the pig EBV data.

Layer (type)	Output shape	# Parameters
Conv1d (Conv1D)	(None, 23657, 41)	1681
Maxpooling (MaxPooling1D)	(None, 11828, 41)	0
Flatten (Flatten)	(None, 484948)	0
Out (Dense)	(None, 1)	484949
Total		486630

The model description is based on the output from the summary() function in Keras. None is an internal Keras representation which means that the mini-batch size is a variable to be set in the consequent fit() function.

**Table 4:** Layer name and type, output data shape of the layer, and number of trainable parameters per layer for the model with the Bayesian optimized model averaged hyperparameters using the pig raw phenotype data.

Layer (type)	Output shape	# Parameters
Conv1d (Conv1D)	(None, 23650, 25)	875
Maxpooling (MaxPooling1D)	(None, 11825, 25)	0
Flatten (Flatten)	(None, 295625)	0
Out (Dense)	(None, 1)	295626
Total		296501

The model description is based on the output from the summary() function in Keras. None is an internal Keras representation which means that the mini-batch size is a variable to be set in the consequent fit() function.

**Figure 2:**
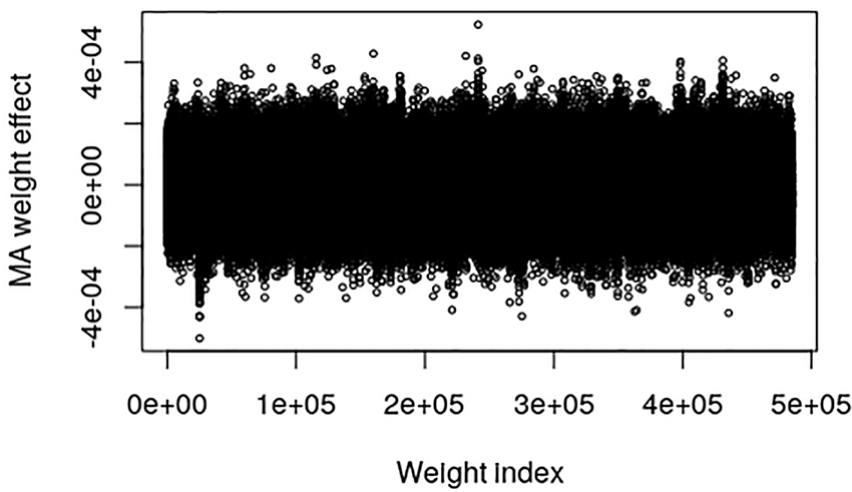
Plot of model averaged weight effects against weight index in the output layer when breeding values (EBVs) of the Austrian pig data are used as phenotypes.

**Figure 3:**
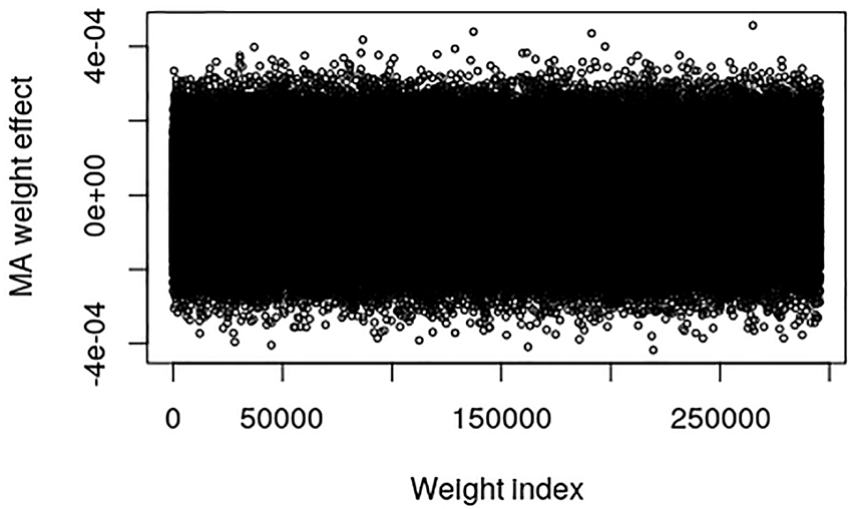
Plot of model averaged weight effects against weight index in the output layer when number of live born piglet of the Austrian pig data are used as phenotypes.

The pig data contains no known QTL positions. The mapping factor is equal to 484,949/47,299 = 10.2 and 295,626/47,299 = 6.25 for the EBV and raw phenotype, respectively. **Figure 2** provides a plot of the MA weight effects of the output layer against weight index for the EBV. There are some indications of QTL-peaks, especially one with negative effects around weight index 25,000 which maps back to SNP 2450. No peaks can be found for the raw phenotype, which is not surprising because of the low heritability (**Figure 3**).

## Discussion

Recently, Waldmann (2018) showed how the dropout technique can be applied to an MLP, resulting in an approximate Bayesian (ABNN) model that provided a computationally efficient approach. The ABNN method was compared with GBLUP and BLASSO on the same simulated dataset used in the present study. The resulting testing set MSE on the simulated QTLMAS2010 data was 82.69, 88.42, and 89.22 for the ABNN, GBLUP, and BLASSO, respectively. Waldmann (2018) used the Cleveland pig data (Cleveland et al., 2012) as an example of a real data and found the test MSE estimates were equal to 0.865, 0.876, and 0.874 for ABNN, GBLUP, and BLASSO, respectively. Unfortunately, the Cleveland data could not be used in the current study because the order of the SNPs was randomized which breaks down the LD structure.

There are some interesting properties and findings of the CNNGWP worth mentioning. In contrast to the ABNN (Waldmann, 2018), dropout did not improve on prediction error. The reason for this is probably that the *ℓ*_1_-norm constraint of the weights in the output layer of the CNNGWP is equivalent to the LASSO, which is known to be a more efficient regularizer. Another consideration is that the linear activation function was superior to the ReLU activation, irrespective of whether the latter was applied directly to the one-dimensional convolution layer or in an extra fully connected layer before the pooling layer. Hence, there is not much non-linearity to capture by the model apart from the dominance, which is not surprising because of the 0, 1, and 2 coding of the SNPs. It can also be seen in the plot of the weights in the output layer of the QTLMAS2010 data that CNNGWP captures the true QTLs with clear peaks that indicate whether a region is mostly additive or dominant. The difference in the plots of the weights of the real pig data is most likely caused by the the fact that the EBV phenotype has less error variance than the raw phenotype.

Regarding computational efficiency, it is difficult to give an exact timing of the CNNGWP analyses because it depends heavily on what strategies that are used for parallel computing, the need for initial ad-hoc evaluation of hyper-parameters, the number of epochs in ADAM, and number of iterations and replicates of the BO. The most computationally demanding part is the BO of the hyper-parameters because of the sequential nature of this algorithm. Speed ups can be obtained by using the GPU version of Keras (Chollet et al., 2017), and by fully parallelizing the BO and ensemble prediction. With a carefully optimized strategy it would be possible to appraoch a computing time of a few hours of based on data sets of similar size as in this study.

The dominant design of the relatively few applications of NN in GWP is the MLP (e.g., Ehret et al. (2015) and Glória et al. (2016)). Bellot et al. (2018) compared deep learning models on large human SNP data combined with five phenotypes with varying levels of heritability. For height, a highly heritable phenotype, all methods performed more or less similarly, although CNNs were slightly but consistently worse. For the rest of the phenotypes, the performance of some CNNs was comparable to, or slightly better than, that of Bayesian linear methods. Performance of MLPs was highly dependent on SNP set and phenotype. Over the range of traits evaluated in that study, CNN performance was competitive with that of linear models, but the difference from the linear model was not large in any of the cases investigated. The CNN models in that study have some similarities with CNNGWP, but the authors regularized weight parameters with dropout, used genetic algorithms for hyperparameter tuning, and did not use model averaged ensemble prediction. Bellot et al. (2018) also showed that CNNs performed comparatively better as narrow-sense heritability decreased and the contribution of dominance increased. These results are consistent with those obtained with the CNNGWP analysis of the simulated QTLMAS2010 data. Our conclusion is that CNNGWP and related CNN approaches can improve prediction accuracy, but the magnitude of improvement most likely depends on the genetic architecture and to some extent on the heritability. A thourough analysis of simulated data sets with different genetic architechtures and heritabilities would be needed to shed more light on this issue.

Ma et al. (2018) proposed a deep convolutional NN (DeepGS) with a fixed 8–32–1 architecture including ReLU activation and dropout between all layers. They evaluated prediction performance as the phenotypic values of the eight traits scored on 2,000 individuals of wheat and 33,709 DArT (Diversity Array Technology) markers. In general, they found that DeepGS performed only marginally better than GBLUP and RR-BLUP, but considerably better than three different MLPs. However, the lack of formal hyperparameter and NN structure optimization limits the usefulness of that result.

There is increasing interest in applications of deep learning in various areas of genomics (Zou et al., 2019). However, most of the applications concern functional genomics, with examples including predicting the sequence specificity of DNA- and RNA-binding proteins and of enhancer and cis-regulatory regions, methylation status, gene expression, and control of splicing. Deep learning has been especially successful when applied to regulatory genomics, by using architectures directly adapted from modern computer vision and natural language-processing applications. Some ongoing work is focusing on predicting phenotypes from genetic data, but most of these examples concern tools for base calling and structural prediction. In the future, it will be interesting to see if integration of data from different sources, for example DNA variants, RNA expression profiles, other types of omics data as well as environmental variables can contribute to better prediction accuracy (Grapov et al., 2018).

Finally, we would like to provide some notes regarding the importance of modeling of the spatial structure between the genomic markers. A large number of methods have been developed for incorporation of LD or physical marker order. Yang and Tempelman (2012) suggested Bayesian first-order antedependence models and reported that these methods had higher accuracies compared with models without the antedependence parameters between the markers. Recently, Wang et al. (2019) devloped the Precision Lasso, which is a Lasso variant that promotes sparse variable selection by regularization directed by the covariance and inverse covariance matrices of the explanatory variables. They found that the variable selection properties improved, but the prediction error increased. It has also been proposed to model the marker dependency between the markers using different graph procedures (Martinez et al., 2017; Stephenson et al., 2019). A unifying factor for all these approaches is that the spatial structure is used to regularize and/or smooth the predictors, but the number of predictors is constant through the model. In contrast, the convolution operator in CNNGWP first expands the number of parameters locally, and then regularizes these *via* the maxout pooling and the *ℓ*_1_-norm on the output layer. Further work is definitely needed in this area, both on development of new methods and to establish mathematical relationships between all these methods.

## Data Availability Statement

The simulated dataset analyzed in this study can be found at github https://github.com/patwa67/CNNGWP. The pig data that support the findings of this study are available from University of Natural Resources and Life Sciences Vienna (BOKU) but restrictions apply to the availability of these data, which were used under license for the current study, and so are not publicly available. Data are however available from the authors upon reasonable request and with permission of University of Natural Resources and Life Sciences Vienna (BOKU).

## Ethics Statement

Ethical review and approval was not required for the animal study because the study was performed with data from production animals.

## Author Contributions

All the authors contributed to the method design. CP and GM acquired and edited the Austrian Large White and Landrace sow data. PW implemented the algorithm, carried out the experiments, and wrote the paper. All authors read and approved the final manuscript.

## Funding

Financial support was provided by the Beijer laboratory for animal science, SLU, Uppsala.

## Conflict of Interest

The authors declare that the research was conducted in the absence of any commercial or financial relationships that could be construed as a potential conflict of interest.
